# Osmoregulated Periplasmic Glucans Transmit External Signals Through Rcs Phosphorelay Pathway in *Yersinia enterocolitica*

**DOI:** 10.3389/fmicb.2020.00122

**Published:** 2020-02-05

**Authors:** Jiao Meng, Can Huang, Xiaoning Huang, Dingyu Liu, Beizhong Han, Jingyu Chen

**Affiliations:** ^1^Beijing Laboratory for Food Quality and Safety, College of Food Science and Nutritional Engineering, China Agricultural University, Beijing, China; ^2^Beijing Advanced Innovation Center for Food Nutrition and Human Health, College of Food Science and Nutritional Engineering, China Agricultural University, Beijing, China; ^3^Department of Biochemical Engineering, School of Chemical Engineering and Technology, Tianjin University, Tianjin, China

**Keywords:** *Yersinia enterocolitica*, osmoregulated periplasmic glucans, Rcs phosphorelay, EnvZ/OmpR phosphorelay, gene expression, pathogenic phenotype

## Abstract

Fast response to environmental changes plays a key role in the transmission and pathogenesis of *Yersinia enterocolitica*. Osmoregulated periplasmic glucans (OPGs) are known to be involved in environmental perception of several *Enterobacteriaceae* pathogens; however, the biological function of OPGs in *Y. enterocolitica* is still unclear. In this study, we investigated the role of OPGs in *Y. enterocolitica* by deleting the *opgGH* operon encoding enzymes responsible for OPGs biosynthesis. Complete loss of OPGs in the Δ*opgGH* mutant resulted in decreased motility, c-di-GMP production, biofilm formation and smaller cell size, whereas the overproduction of OPGs through restoration of *opgGH* expression promoted c-di-GMP/biofilm production and increased antibiotic resistance of *Y. enterocolitica*. Gene expression analysis revealed that *opgGH* deletion reduced transcription of *flhDC*, *ftsAZ*, *hmsT* and *hmsHFRS* genes regulated by the Rcs phosphorelay system, whereas additional deletion of *rcs* family genes (*rcsF*, *rcsC*, or *rcsB*) reversed this effect and restored motility and c-di-GMP/biofilm production but further reduced cell size. Furthermore, disruption of the Rcs phosphorelay increased the motility and promoted the induction of biofilm and c-di-GMP production regulated by OPGs through upregulating the expression of *flhDC*, *hmsHFRS*, and *hmsT*. However, deletion of genes encoding the EnvZ/OmpR phosphorelay downregulated the *flhDC*, *hmsHFRS* and *hmsT* expression, leading to the decreased motility and prevented the induction of biofilm and c-di-GMP production regulated by OPGs. These results indicated that Rcs phosphorelay had the effect on OPGs-mediated functional responses in *Y. enterocolitica*. Our findings disclose part of the biological role of OPGs and the underlying molecular mechanisms associated with Rcs system in the regulation of the pathogenic phenotype in *Y. enterocolitica.*

## Introduction

*Yersinia enterocolitica* is a common foodborne pathogen which causes a zoonotic disease called yersiniosis manifested in humans by acute gastroenteritis and sometimes more serious conditions such as pseudoappendicitis and even sepsis (Huovinen et al., 2010; Bari et al., 2011; Fabrega and Vila, 2012). In the reports of the European Food Safety Authority (EFSA), *Y. enterocolitica* is listed as the third most common enteropathogen after *Campylobacter* and *Salmonella* (Zadernowska et al., 2014). *Y. enterocolitica* is widely distributed in the environment and can be found in soil, water, animals and various food products (Bari et al., 2011; Rahman et al., 2011), where constant changes in physicochemical conditions, including osmolarity, pH, temperature, light intensity, medium viscosity, and nutrient availability often threaten bacterial survival (Bottone, 1997; Brzostek et al., 2012). These challenges are met by rapid adaptation of the pathogen to varying growth conditions provided by two-component regulatory systems (TCSs), also called phosphorelays, as they sense the extracellular signals and perform cascade phosphorylation in response, thus regulating the expression of genes related to flagellar synthesis, biofilm formation, and virulence (Clarke, 2010; Clarke and Voigt, 2011; Brzostek et al., 2012; Liu et al., 2017).

EnvZ/OmpR is a classical TCS present in many bacteria, including *Y. enterocolitica* (Brzostek et al., 2012). Under environmental stimuli, transmembrane histidine kinase EnvZ (sensor) is autophosphorylated and in turn phosphorylates a transcription factor OmpR, which regulates the expression of genes controlling numerous bacterial cell functions, including outer membrane permeability (Russo and Silhavy, 1991), flagella synthesis (Raczkowska et al., 2011), biofilm formation (Pruss, 2017), and pH tolerance (Bang et al., 2000). It has been reported that in *Y. enterocolitica*, OmpR could play an important role in controlling the virulence properties (Dorrell et al., 1998; Brzostek et al., 2003), act as the response regulator for osmolarity-regulated porins and Yop proteins (Brzostek et al., 2003), negatively regulate invasion gene expression (Brzostek et al., 2007) and positively control motility and *flhDC* expression (Raczkowska et al., 2011). The EnvZ/OmpR phosphorelay has been reported to be a central regulation system of several cellular responses in *Y. enterocolitica* (Brzostek et al., 2012).

The regulator of capsule synthesis (Rcs) phosphorelay is an atypical TCS restricted to Enterobacteria (Wall et al., 2018). This system is composed of three core proteins, the transmembrane sensor kinase RcsC, transmembrane protein RcsD, and response regulator RcsB, and could be activated by an outer membrane-associated lipoprotein RcsF in response to stress (Castanie-Cornet et al., 2006). As a result of this phosphorelay, RcsB is phosphorylated and then interacts with a conserved motif in target genes, thus regulating their transcription (Clarke, 2010). In several Enterobacteria spp., the Rcs system downregulates the *flhDC* master operon encoding activators required for expression of the flagellar apparatus genes and upregulates the *ftsAZ* operon needed for cell division (Bontemps-Gallo et al., 2013). A recent study reported that in *Yersinia pestis*, the Rcs system inhibited biosynthesis of cyclic dimeric guanosine monophosphate (c-di-GMP) and biofilm formation by directly repressing transcription of the *hmsT* gene and the *hmsHFRS* operon (Fang et al., 2015). The Rcs system has also been reported in *Y. enterocolitica*; however, the signaling mechanism underlying its participation in environmental responses is still unclear.

Osmoregulated periplasmic glucans (OPGs) formerly known as membrane-derived oligosaccharides are polymers of D-glucose connected through β-linkage (Bontemps-Gallo and Lacroix, 2015), which are present in the cell envelope of Gram-negative bacteria (Bontemps-Gallo et al., 2017). As the name indicates, OPGs are osmoregulated and their synthesis and accumulation decreases with the increase in osmolarity (Bhagwat et al., 2009; Bontemps-Gallo et al., 2013). The synthesis of OPGs requires the *opgGH* operon in Enterobacteria or its functional homologs *ndvAB/chvAB/cgs* in other bacterial species (Bontemps-Gallo et al., 2017). Deletion of these operons causes a total loss of OPGs, resulting in a pleiotropic phenotype (Bontemps-Gallo and Lacroix, 2015). OPGs represent virulence factors of many pathogenic bacteria; thus, *Dickeya dadantii* and *Salmonella* lacking OPGs exhibited a non-virulent phenotype (Page et al., 2001; Bhagwat et al., 2009). In *Escherichia coli*, OPGs were shown to regulate chemotaxis, motility and intercellular signaling (Weissborn et al., 1992), whereas in *Brucella abortus*, OPGs secretion was required in the early stage of cell infection and invasion (Briones et al., 2001), and in *Pseudomonas aeruginosa*, the presence of OPGs facilitated biofilm formation and conferred higher antibiotic resistance (Mah et al., 2003). Furthermore, it has been reported that mutations in the operon responsible for structural assembly of OPGs led to activation of the Rcs phosphorelay. Thus, in *E. coli*, the lack of OPGs was reported to trigger the Rcs phosphorelay, which resulted in suppression of bacterial motility (Girgis et al., 2007), whereas OPGs-negative *D. dadantii* totally lost virulence and motility, which was due to constitutive activation of the Rcs phosphorelay (Bouchart et al., 2010; Bontemps-Gallo et al., 2013; Madec et al., 2014). These data indicate that OPGs play an important role in the pleiotropic phenotype of pathogenic bacteria, showing a regulatory effect on TCSs.

As a member of the *Enterobacteriaceae* family, *Y. enterocolitica* also contains the complete *opgGH* gene cluster as revealed by genome-wide sequencing (Thomson et al., 2006), but the functional significance of *opgGH* in *Y. enterocolitica* is largely unknown; the only evidence of it is that OpgH is required in the early stage of infection (Young and Miller, 1997). Except for *Y*. *enterocolitica*, there are other two human pathogenic species in the genus *Yersinia*, *Yersinia pseudotuberculosis*, and *Y. pestis* (Kim et al., 2008). *Y. pseudotuberculosis* causes gastroenteritis, while *Y. pestis* is the causative agent of plague. Recent studies found that *Y. pseudotuberculosis* 2777 (serotype O:1) and 2515 (serotype O:2) did not produce OPGs. With the deletion of *opgGH*, *Y. pseudotuberculosis* showed normal motility, biofilm formation and virulence, but smaller cell size (Quintard et al., 2015). *Y. pestis* lost the *opgGH* operon during its emergence from *Y. pseudotuberculosis*. After re-introducing *opgGH* into *Y. pestis*, no effect on flea proventricular blockage rate was found (Quintard et al., 2015). All these studies on the biological functions of *opgGH* in Enterobacteria spp. led us to explore the role of *opgGH* and its products in *Y. enterocolitica*.

In this study, we showed that OPGs were present in *Y. enterocolitica* (biotype 1B and serotype O:8) and that deletion of the *opgGH* operon generated a pleiotropic phenotype. Gene expression analysis indicated that OPGs deficiency was correlated with the activation of the Rcs phosphorelay, whereas OPGs overexpression enhanced c-di-GMP production and biofilm formation and conferred higher antibiotic resistance to *Y. enterocolitica*. It was also found that Rcs and EnvZ/OmpR phosphorelays had opposite effects on the regulation of OPGs-induced c-di-GMP production and biofilm formation in *Y. enterocolitica*. These findings disclose part of the biological role of OPGs and further understanding of Rcs and EnvZ/OmpR phosphorelays in the regulation of the pathogenic phenotype in *Y. enterocolitica.*

## Materials and Methods

### Bacterial Strains and Culture Conditions

Bacterial strains and plasmids used in this study are listed in Table 1. *E. coli* DH5α used as the host bacteria in plasmid construction was cultured at 37°C in lysogeny broth (LB) consisting of 5 g/L yeast extract, 10 g/L tryptone, and 5 g/L NaCl. *Y. enterocolitica* ATCC23715 (biotype 1B and serotype O:8) was used as the parent strain for construction of *Y. enterocolitica* mutants; the bacteria were cultured at 26°C in different growth media: LB, LBNS (LB without salts) and LNNS (LBNS broth diluted 1:8 in distilled water). Ampicillin (100 μg/ml), chloramphenicol (16 μg/ml), cefsulodin (15 μg/ml), irgasan (4 μg/ml), and novobiocin (2.5 μg/ml) were added as required.

**TABLE 1:** Strains and plasmids used in this study.

Strains and plasmids	Relevant characteristics	Sources
***Y. enterocolitica***		
ATCC23715	WT, serotype O:8, Biotype 1B, pYV^–^	Lab stock
YE-1	△*opgGH*	This study
YE-2	△*opgGH*, P_*BAD*_*opgGH*; Amp^*r*^	This study
YE-B	△*rcsB*	Meng et al., 2019
YE-C	△*rcsC*	This study
YE-F	△*rcsF*	This study
YE-1B	△*opgGH*, △*rcsB*	This study
YE-1C	△*opgGH*, △*rcsC*	This study
YE-1F	△*opgGH*, △*rcsF*	This study
YE-2B	△*opgGH*, △*rcsB*, P_*BAD*_*opgGH*; Amp^*r*^	This study
YE-2C	△*opgGH*, △*rcsC*, P_*BAD*_*opgGH*; Amp^*r*^	This study
YE-2F	△*opgGH*, △*rcsF*, P_*BAD*_*opgGH*; Amp^*r*^	This study
YE-Z	△*envZ*	This study
YE-R	△*ompR*	Meng et al., 2019
YE-1Z	△*opgGH*, △*envZ*	This study
YE-1R	△*opgGH*, △*ompR*	This study
YE-2Z	△*opgGH*, △*envZ*, P_*BAD*_*opgGH*; Amp^*r*^	This study
YE-2R	△*opgGH*, △*ompR*, P_*BAD*_*opgGH*; Amp^*r*^	This study
***E. coli***		
S17-1 λpir	*recA1*, *thi*, *pro*, *hsdR*-M ^+^, RP4:2-Tc:Mu^–^Kan:Tn*7*, λpir	Lab stock
DH5a	F^–^, φ80*lacZ*△*M15*, △(*lacZYA-argF*)*U169*, *deoR*, *recA1*, *endA1*, *hsdR17* (rk^–^,mk^+^), *phoA*, *supE44*, λ^–^, *thi-1*, *gyrA96*, *relA1*	Lab stock
**Plasmids**		
pDS132	Conditional replication vector;	Lab stock
	R6K origin, mobRK4 transfer origin, sucrose-inducible-*sacB*; Cm^*r*^	
pDS132-△*opgGH*	Upstream and downstream *opgGH* fragments	This study
	were cloned into pDS132; Cm^*r*^	
pDS132-△*rcsB*	Upstream and downstream *rcsB* fragments	This study
	were cloned into pDS132; Cm^*r*^	
pDS132-△*rcsC*	Upstream and downstream *rcsC* fragments	This study
	were cloned into pDS132; Cm^*r*^	
pDS132-△*rcsF*	Upstream and downstream *rcsF* fragments	This study
	were cloned into pDS132; Cm^*r*^	
pDS132-△*envZ*	Upstream and downstream *envZ* fragments	This study
	were cloned into pDS132; Cm^*r*^	
pDS132-△*ompR*	Upstream and downstream *ompR* fragments	This study
	were cloned into pDS132; Cm^*r*^	
pBAD24	*AraC*, promoter P_*BAD*_; Amp^*r*^	Lab stock
pBAD24-*opgGH*	*AraC*, P_*BAD*_ *opgGH;* Amp^*r*^	This study

**Amp*, ampicillin; *Cm*, chloramphenicol; *r*, resistance.*

### Plasmid Construction

To construct pDS132-Δ*opgGH*, fragments upstream and downstream of the *opgGH* gene were amplified from the *Y. enterocolitica* genome using primers *opgGH-up-F/opgGH-up-R* and *opgGH-down-F/opgGH-down-R.* The upstream and downstream fragments were fused and amplified by fusion PCR with primers *opgGH-up-F/opgGH-down-R*; the resultant long fragment was digested with *Sph*I and *Sac*I and ligated into pDS132 digested with the same enzymes to yield pDS132- Δ*opgGH*. The same approach was also used to construct pDS132-Δ*rcsF*, pDS132-Δ*rcsC*, pDS132-Δ*rcsB*, pDS132-Δ*envZ*, and pDS132-Δ*ompR* with the corresponding primers.

To construct pBAD24-*opgGH*, the *opgGH* fragment was amplified from the *Y. enterocolitica* genome using primers *p-opgGH-F*/*p-opgGH-R*, digested with *Sal*I and *Hin*dIII, and inserted into pBAD24 digested with the same enzymes. The resultant pBAD24-*opgGH* plasmid contained the *opgGH* operon controlled by the *araBAD* promoter (P_*BAD*_*opgGH*).

### Strain Construction

For construction of the *opgGH* knockout strain, the suicide plasmid pDS132-Δ*opgGH* was introduced into *E. coli* S17-1λpir by electroporation and then mobilized into *Y. enterocolitica* by conjugation. The strategy used for gene deletion in the *Y. enterocolitica* chromosome was based on the two-step homologous recombination procedure described previously (Schafer et al., 1994); the process of genetic manipulation is shown in Supplementary Figure 1. The same approach was used for deletion of the *rcsF*, *rcsC*, *rcsB*, *envZ*, and *ompR* genes in the Δ*opgGH* mutant and wild-type strain, respectively. The mutants were verified by polymerase chain reaction (PCR) and further confirmed by gene sequencing. The pBAD24-*opgGH* plasmid was used to transform Δ*opgGH*, Δ*opgGH-*Δ*rcsF*, Δ*opgGH-*Δ*rcsC*, Δ*opgGH-*Δ*rcsB*,Δ*opgGH-*Δ*envZ*, and Δ*opgGH-*Δ*ompR* mutant strains by electroporation to yield Δ*opgGH*-P_*BAD*_*opg GH*, Δ*opgGH-*Δ*rcsF*-P_*BAD*_*opgGH*, Δ*opgGH*-Δ*rcsC-*P_*BAD*_*opgGH*, Δ*opgGH-*Δ*rcsB*-P_*BAD*_*opgGH*,*ΔopgGH*-Δ*envZ-*P_*BAD*_*opgGH*, and Δ*opgGH-*Δ*ompR-*P_*BAD*_*opgGH* strains. All primers used for strain and plasmid construction are listed in Supplementary Table 1.

### Large-Scale Purification and Determination of OPGs

Osmoregulated periplasmic glucans extraction, purification, and determination were based on the method as described previously (Bontemps-Gallo et al., 2013). To maximize the yield of OPGs, bacteria were grown in 8 L of LNNS medium supplemented with 0.6 g/L L-arabinose until the exponential phase, collected by centrifugation at 8,000 × *g* for 15 min at 4°C, resuspended in 200 ml of distilled water, and treated with 5% trichloroacetic acid. After centrifugation at 8,000 × *g* for 15 min at 4°C, the supernatant was collected and OPGs were extracted by charcoal adsorption, eluted with 15% aqueous pyridine, concentrated by rotary evaporation to a volume of 2 ml, and fractionated by gel filtration on a Bio-Gel P-4 column (1.6 cm × 55 cm; Bio-Rad). OPGs were eluted with 0.5% acetic acid at a flow rate of 15 ml/h, and 1.5-ml fractions were collected. Fractions containing OPGs were pooled and OPGs content was determined by a colorimetric method using the anthrone reagent (Spiro, 1966). Whole-cell protein amount was determined using the BCA Protein Assay Kit (Thermo Fisher Scientific). Final OPGs content was expressed as μg/mg of protein.

### Growth Assay

Growth rates of wild-type, Δ*opgGH* mutant and Δ*opgGH*-P_*BAD*_*opgGH* strains were determined in LB, LBNS, and LNNS media. Single colonies were inoculated into appropriate medium and incubated overnight at 26°C with shaking. The starter cultures were diluted in 100 ml medium to OD_600_ about 0.05 and incubated in 250-ml flasks in a rotatory shaker at 180 rpm. Cell growth was measured at 600 nm in a spectrophotometer (Puxi Universal, Co., Ltd., Beijing).

### Motility Assay

Swim motility experiments were performed in LNNS plates containing 0.35% agar. Bacteria were grown overnight in LB at 26°C, diluted to OD_600_ of 1. And 1 μl of diluted seed cultures were inoculated into swim agar plates and incubated at 26°C. Bacterial swim diameters were measured after 48-h incubation.

### Biofilm Assay

Overnight cultures of *Y. enterocolitica* strains were inoculated into a 96-well plate containing 200 μl LNNS medium per well (initial OD_600_ about 0.05) and incubated at 26°C; six replicates per condition were used. Growth medium was renewed every 24 h. After 24, 48 and 72 h of incubation, growth medium was removed, and the formed biofilms were washed twice with PBS and stained with crystal violet staining solution (0.1%). Wells were washed with water and treated with ethanol-acetone solution to release the dye absorbed in the biofilm, which was then measured at 595 nm.

### Measurement of Bacterial Cell Size

Bacteria grown to the mid-log phase in LNNS medium were immobilized on 200-mesh copper grids, fixed in 0.5% glutaraldehyde for 5 min, washed with water and air-dried. And the cell length was visualized and measured using a JEM-1230 transmission electron microscopy. Bacterial cells were magnified 10,000 times with approximately 30–50 cells per field of view. The cell length of each strain was the average of 100 measurements performed using iTEM software.

### Extraction and Quantification of c-di-GMP

Bacteria were grown to the exponential phase in LNNS medium and 2 ml of planktonic culture with OD_600_ about 0.5 was centrifuged at 10,000 × *g* for 10 min at 4°C. The cell pellet was washed twice with ice-cold PBS, resuspended in 2 ml ice-cold PBS, incubated at 100°C for 5 min, and sonicated for 15 min (power 100%, frequency 37 kHz) in an ice-water bath. After centrifugation, the supernatant containing extracted c-di-GMP was collected and the pellet was resuspended in 2 ml ice-cold PBS; the extraction procedure described above was repeated twice. The extracts were concentrated by cooling evaporation at 4°C to a volume of 500 μl and intracellular c-di-GMP levels were determined by the c-di-GMP enzyme-linked immunosorbent assay (ELISA) Kit (Mskbio, Beijing, China). Cell protein was determined by the BCA assay and c-di-GMP concentrations were expressed as pmol/mg of protein.

### Antibiotic Susceptibility Assay

The sensitivity of *Y. enterocolitica* strains to chloramphenicol and polymyxin B was determined by the agar disk diffusion method as described by Clinical and Laboratory Standards Institute (CLSI, 2012) with some modifications. Briefly, indicated strains were grown to 0.5 McFarland standard and 200 μl of bacterial suspension was streaked over LNNS agar using sterile cotton swabs. Paper disks (6 mm in diameter) containing different concentrations of chloramphenicol or polymyxin B were placed in the center of the plate and the diameter of the clear zone around the disk was measured after 24-h incubation at 26°C.

### RNA Extraction and Real-Time Quantitative PCR (RT-qPCR)

Total RNA was extracted from *Y. enterocolitica* strains grown to the exponential phase in LNNS medium using the TransZol Up Plus RNA Kit (TransGen, Beijing, China). The extracted RNA was then tested for its concentration and quality using a Nanodrop 2000c (Thermo). And cDNA was synthesized from 500 ng RNA using Quant Reverse Transcriptase in the presence of random primers (TransGen, Beijing, China). RT-qPCR was carried out using Real Master Mix (SYBR Green) and specific primers (Supplementary Table 2) in a Light Cycler 480 II (Roche, Basel, Switzerland) under the following cycling conditions: 5 min at 50°C, 30 s at 94°C, and 45 cycles of 5 s at 94°C, and 30 s at 60°C. Reactions were performed in triplicate. Relative transcription of the target genes was analyzed by the 2^–Δ^ ^Δ^ ^*Ct*^ method described previously (Meng et al., 2016); the 16S rRNA gene was used as reference for normalization.

### Statistical Analysis

One-way analysis of variance was performed in SPSS for Windows 20.0 (SPSS, Inc., Chicago, IL, United States).

## Results

### OPGs Were Required for *Y. enterocolitica* Growth in Low-Nutrient Low-Salt Medium

**FIGURE 1:**
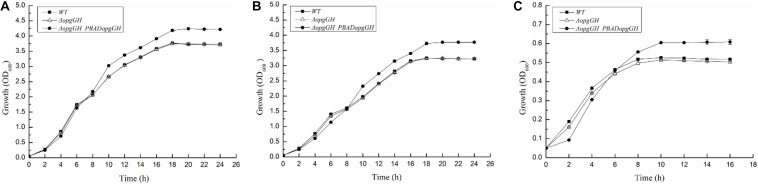
Growth characteristics of the wild-type, Δ*opgGH* and Δ*opgGH*-P_*BAD*_*opgGH* strains of *Yersinia enterocolitica.* Bacteria were grown in LB **(A)**, LBNS **(B)**, and LNNS **(C)** media supplemented with 0.6 g/L L-arabinose. The data are presented as the mean ± SD of at least three independent experiments.

It has been reported that OPGs take part in environmental perception, and their abundance in the periplasm increases as the osmolarity of the medium decreases (Bhagwat et al., 2009; Bontemps-Gallo et al., 2013). Lack of OPGs has been confirmed to impair the optimal growth potential of *Salmonella enterica* serovar Typhimurium under low osmolarity conditions (Bhagwat et al., 2009). To determine the biological function of OPGs in *Y. enterocolitica*, we constructed an OPGs-deficient strain by deleting the *opgGH* operon responsible for OPGs synthesis from the *Y. enterocolitica* chromosome, and an OPGs-overproducing strain by transforming the Δ*opgGH* mutant with the pBAD24-*opgGH* plasmid containing the *opgGH* operon under the control of the inducible P_*BAD*_ promoter. Expression levels of the *opgG* and *opgH* genes were upregulated by 605- and 474-fold, respectively, after addition of 0.6 g/L L-arabinose, indicating that the *opgGH* operon was upregulated by L-arabinose. To assess the effect of osmotic stress and nutrient deficiency on *Y. enterocolitica* growth, we measured growth characteristics of wild-type, Δ*opgGH* and Δ*opgGH*-P_*BAD*_*opgGH* strains in LB, LBNS (LB, no salts) and LNNS (low nutrient, no salts) media. All tested strains were supplemented with 0.6 g/L L-arabinose. The deletion of *opgGH* did not affect bacterial growth in LB and LBNS, but caused a slight lag throughout the growth process in LNNS medium (*P* < 0.05), whereas *opgGH* overexpression led to cells reaching a significantly higher density in stationary phase in LB, LBNS, and LNNS media compared to wild-type strain (*P* < 0.01) (Figures 1A–C).

**FIGURE 2:**
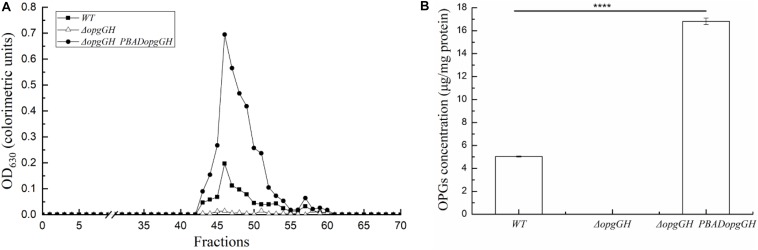
Purification and quantification of OPGs from *Y. enterocolitica*. OPGs were extracted from wild-type, Δ*opgGH* and Δ*opgGH*-P_*BAD*_*opgGH* strains grown to the exponential phase in LNNS medium supplemented with 0.6 g/L L-arabinose. **(A)** Gel filtration chromatography of OPGs. **(B)** Quantification of purified OPGs. The data are presented as the mean ± SD of at least three independent experiments. An asterisk indicates a significant difference with ****P < 0.0001.

Deletion of *opgGH* showed impaired cell growth in LNNS medium, indicating that the growth of wild-type *Y. enterocolitica* was relatively dependent of OPGs synthesis under low-nutrient low-salt conditions. Then, we performed extraction and separation of OPGs from *Y. enterocolitica* cultured in LNNS medium. OPGs from wild-type and Δ*opgGH*-P_*BAD*_*opgGH* strains were eluted from the BioGel P4 gel filtration column as a single major peak (fractions 43–55), whereas the elution profile of the Δ*opgGH* mutant lacked the corresponding peak (Figure 2A), suggesting the absence of OPGs in the Δ*opgGH* strain. Concentrations of OPGs in wild-type and Δ*opgGH*-P_*BAD*_*opgGH* strains were 5.03 and 16.81 μg/mg protein, respectively (Figure 2B). This finding, in combination with growth characteristics, indicates that OPGs synthesized by *opgGH*-encoded enzymes regulate the growth of *Y. enterocolitica* under low-nutrient low-salt condition.

### *OpgGH* Deletion Decreased *Y. enterocolitica* Motility, c-di-GMP Production, Biofilm Formation, and Cell Size

**FIGURE 3:**
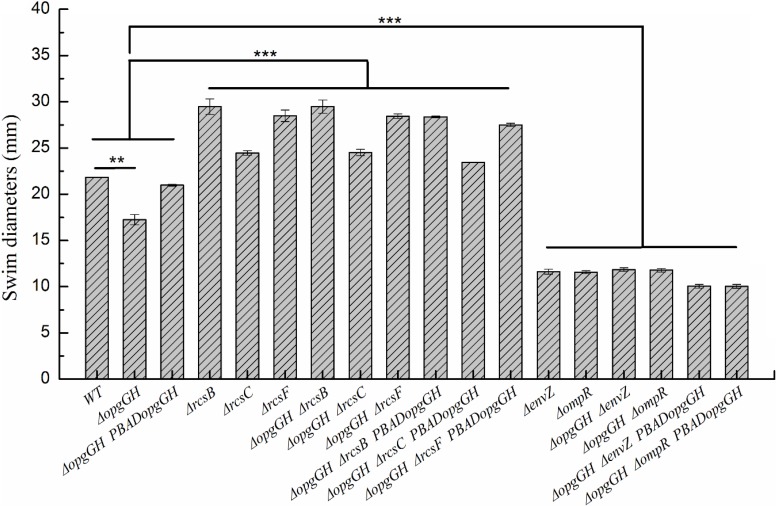
Changes in swim motility of wild-type and mutant strains in LNNS medium. *Y. enterocolitica* was grown in LNNS semisolid plates supplemented with 0.6 g/L L-arabinose at 26°C. Swim diameters were measured after 48 h of incubation. The data are presented as the mean ± SD of at least three independent experiments. An asterisk indicates a significant difference with ***P < 0.001, **P < 0.01.

**FIGURE 4:**
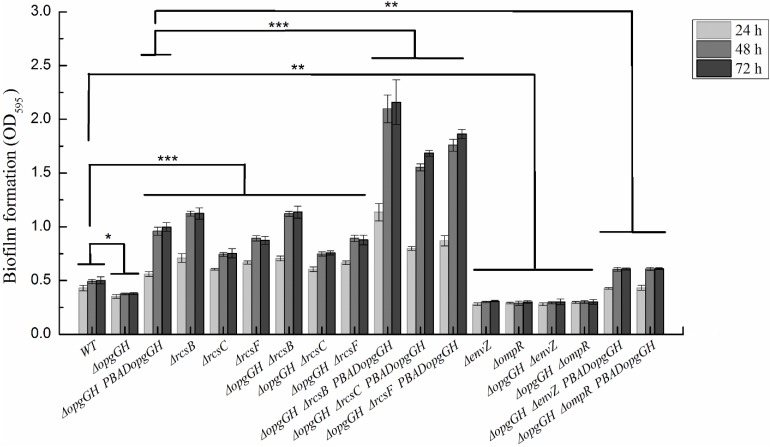
Biofilm formation in wild-type and mutant strains of *Y. enterocolitica*. Bacteria were cultured in LNNS medium supplemented with 0.6 g/L L-arabinose at 26°C. Biofilm formation was analyzed after 24, 48, and 72 h of incubation by staining with crystal violet and measuring absorbance at 595 nm. The data are presented as the mean ± SD of at least three independent experiments. An asterisk indicates a significant difference with ***P < 0.001, **P < 0.01, *P < 0.05.

**FIGURE 5:**
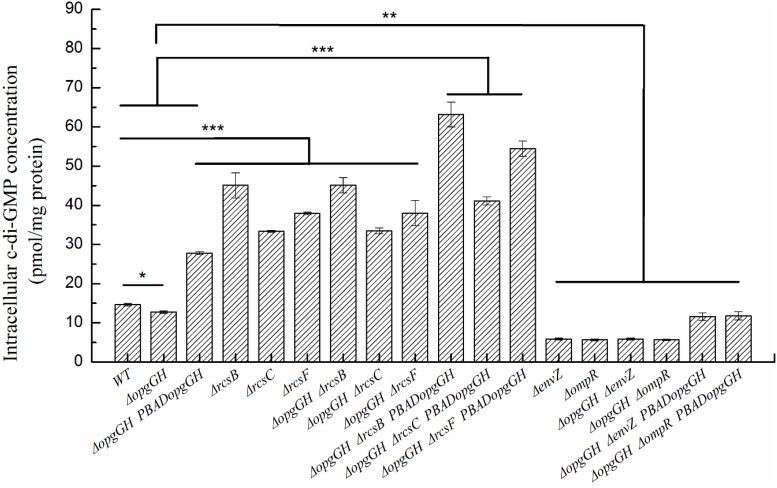
The intracellular c-di-GMP concentration in wild-type and mutant strains of *Y. enterocolitica*. Bacteria were cultured in LNNS medium supplemented with 0.6 g/L L-arabinose at 26°C. Intracellular c-di-GMP levels were analyzed in bacteria grown to the exponential phase. The data are presented as the mean ± SD of at least three independent experiments. An asterisk indicates a significant difference with ***P < 0.001, **P < 0.01, *P < 0.05.

Previous evidence suggests that the lack of OPGs in Proteobacteria caused abnormal phenotypic changes, including decreased envelope stability, flagellar synthesis, biofilm formation, environmental tolerance, virulence, and pathogenicity (Bontemps-Gallo and Lacroix, 2015). Therefore, the effect of loss and overexpression of OPGs on the *Y. enterocolitica* phenotype was analyzed in LNNS medium. The results indicated that the Δ*opgGH* mutant decreased the swim diameter by 21% in LNNS agar, but motility was restored when the mutant was complemented with the wild-type *opgGH* operon (Figure 3 and Supplementary Figure 2). Furthermore, OPGs depletion reduced by 24% in biofilm formation, whereas OPGs overproduction almost doubled the yield of biofilms after 72 h of incubation as evidenced by crystal violet staining when compared to the wild-type strain (Figure 4 and Supplementary Figure 3). Similarly, the absence of OPGs resulted in 13% reduction, while OPGs overproduction led to a 90% increase in c-di-GMP production when compared to the wild-type strain (Figure 5).

**FIGURE 6:**
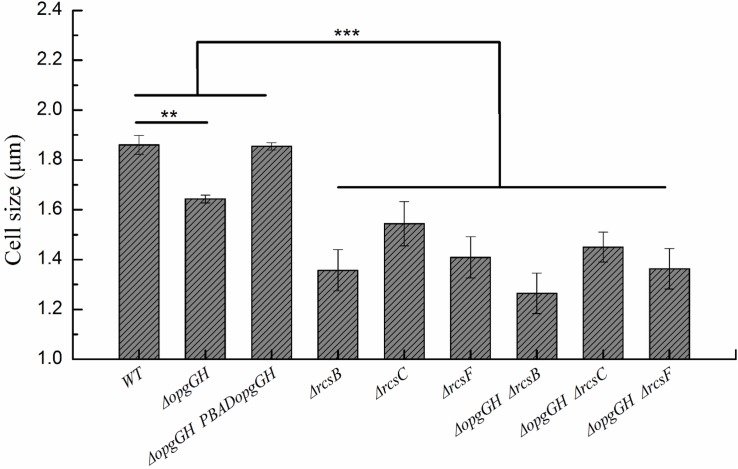
Changes in the cell size of wild-type and mutant strains. Bacteria were grown to the mid-log phase in LNNS medium supplemented with 0.6 g/L L-arabinose and cell size was measured using transmission electron microscopy. The data are presented as the mean ± SD of at least three independent experiments. An asterisk indicates a significant difference with ***P < 0.001, **P < 0.01.

In addition to its ability to synthesize OPGs, the *opgGH* operon (*opgH* but not *opgG*) is also involved in the regulation of bacterial cell size through sequestration of FtsZ, a highly conserved tubulin-like cell division protein (Hill et al., 2013; Quintard et al., 2015). Therefore, we investigated the effect of *opgGH* loss on *Y. enterocolitica* cell shape during growth in LNNS medium. As shown in Figure 6 and Supplementary Figure 4, *opgGH-*deficient cells were about 12% smaller than wild-type cells, but the normal cell size was restored when mutant cells were complemented with *opgGH*, suggesting that *opgGH* (consider as a whole) controls cell size in *Y. enterocolitica.*

### OPGs Overproduction Conferred Higher Antibiotic Resistance to *Y. enterocolitica*

Previous reports suggested that in addition to the presence in the periplasmic space, OPGs can be secreted from the cell and directly interact with antibiotics to protect bacteria and promote their survival (Mah et al., 2003; Bontemps-Gallo and Lacroix, 2015). To investigate the effect of OPGs on antibiotic sensitivity of *Y. enterocolitica*, we assessed the clearing zones produced by chloramphenicol, which inhibits protein synthesis, and polymyxin B, which destabilizes bacterial cell membrane. Visible from the results that *Y. enterocolitica* was more sensitive to chloramphenicol than to polymyxin B (Table 2). For chloramphenicol, both wild-type and Δ*opgGH* mutant strains appeared the clearing areas at a concentration of 0.125 mg/ml, whereas Δ*opgGH*-P_*BAD*_*opgGH* strain were at 0.25 mg/ml. However, 0.25 mg/ml of polymyxin B resulted in the appearance of clearing areas of both wild-type and OPGs-deficient strains, whereas a higher concentration, at 0.5 mg/ml, was required for the emergence of inhibition zones in Δ*opgGH*-P_*BAD*_*opgGH* strain. Further, zone of growth inhibition around a disk was also used to assess the sensitivity to antibiotics. The average sizes of clearing zones at different concentrations of the two antibiotics were slightly larger for the Δ*opgGH* mutant, but were markedly reduced after OPGs overproduction compared to the wild-type strain (*P* < 0.01) (Table 2), suggesting that OPGs increase resistance of *Y. enterocolitica* to at least two different classes of antibiotics.

**TABLE 2:** Susceptibility of *Y. enterocolitica* strains to chloramphenicol and polymyxin B.

Antibiotics	Strains	Average clearing zone (mm)					
		
		0.0625 mg/ml	0.125 mg/ml	0.25 mg/ml	0.5 mg/ml	1 mg/ml	2 mg/ml
Chloramphenicol	WT	−	6.88 ± 0.16	10.20 ± 0.12	13.52 ± 0.10	19.76 ± 0.11	21.48 ± 0.15
	△*opgGH*	−	7.40 ± 0.27	11.04 ± 0.07	14.18 ± 0.29	20.28 ± 0.11	21.81 ± 0.17
	△*opgGH*- P_*BAD*_*opgGH*	−	−	7.55 ± 0.31	10.45 ± 0.31	17.54 ± 0.38	20.47 ± 0.35
Polymyxin B	WT	−	−	1.69 ± 0.10	2.93 ± 0.06	4.68 ± 0.24	5.14 ± 0.17
	△*opgGH*	−	−	2.07 ± 0.04	3.34 ± 0.06	5.00 ± 0.11	5.45 ± 0.10
	△*opgGH*- P_*BAD*_*opgGH*	−	−	−	1.50 ± 0.09	2.71 ± 0.06	3.78 ± 0.05

*The data are presented as the mean ± SD of at least three separate experiments. “−”, not detected.*

### Disruption of the Rcs Phosphorelay in the *Y. enterocolitica* Δ *opgGH* Mutant Restored Motility, c-di-GMP Production, and Biofilm Formation but Further Reduced Cell Size

It has been reported that activation of the Rcs phosphorelay triggered by OPGs deficiency suppressed motility of *E. coli* (Girgis et al., 2007) and attenuated virulence of *D. dadantii* (Bouchart et al., 2010), but the effects were reversed after deletion of any of the Rcs phosphorelay genes. In this study, we constructed Δ*opgGH-*Δ*rcsF*, Δ*opgGH-*Δ*rcsC*, and Δ*opgGH-*Δ*rcsB* double mutants to determine whether disruption of Rcs phosphorelay could reverse the effects caused by OPGs loss in *Y. enterocolitica*. As a result, the double mutants not only restored but showed even better swim motility than the wild-type strain. The Δ*opgGH-*Δ*rcsF*, Δ*opgGH-*Δ*rcsC*, and Δ*opgGH-*Δ*rcsB* double mutants had increased swim diameter by 30, 12, and 35%, respectively, compared to the wild-type strain (Figure 3 and Supplementary Figure 2). In addition, the swim diameter of Δ*rcsF*, Δ*rcsC*, and Δ*rcsB* single mutants were also performed to distinguish the effects of OPGs and Rcs phosphorelay on the motility of *Y. enterocolitica*. Deletion of *rcsF*, *rcsC*, and *rcsB* also increased swim diameter by 31, 12, and 35%, respectively, compared to the wild-type strain (Figure 3 and Supplementary Figure 2). However, there was no significant change in swim diameter between Δ*opgGH-*Δ*rcsF/*Δ*opgGH-*Δ*rcsC/*Δ*opgGH-*Δ*rcsB* double mutant and Δ*rcsF/*Δ*rcsC/*Δ*rcsB* single mutant (*P* > 0.05) (Figure 3), indicating that loss of OPGs will not affect the swim motility in the absence of Rcs phosphorelay in *Y. enterocolitica*.

Similarly, Δ*opgGH-*Δ*rcsF*, Δ*opgGH-*Δ*rcsC*, and Δ*opgGH-*Δ*rcsB* double mutants not only restored but further promoted biofilm and c-di-GMP production compared to the wild-type strain. The biofilm formation in Δ*opgGH-*Δ*rcsF*, Δ*opgGH-*Δ*rcsC*, and Δ*opgGH-*Δ*rcsB* double mutants were 76, 52% and 1.28-fold increase, respectively, compared to the wild-type strain after 72 h of incubation (Figure 4 and Supplementary Figure 3). The intracellular c-di-GMP production in Δ*opgGH-*Δ*rcsF*, Δ*opgGH-*Δ*rcsC*, and Δ*opgGH-*Δ*rcsB* double mutants were 1.59-, 1.28-, and 2.07-fold increase in comparison to the wild-type strain (Figure 5). Although Δ*rcsF*, Δ*rcsC*, and Δ*rcsB* single mutants upregulated the biofilm and c-di-GMP production, there was no significant change between Δ*opgGH-*Δ*rcsF/*Δ*opgGH-*Δ*rcsC/*Δ*opgGH-*Δ*rcsB* double mutant and Δ*rcsF/*Δ*rcsC/*Δ*rcsB* single mutant in terms of biofilm formation and intracellular c-di-GMP concentration (*P* > 0.05) (Figures 4, 5), indicating that loss of OPGs will not affect the biofilm and c-di-GMP production in the absence of Rcs phosphorelay in *Y. enterocolitica*.

However, disruption of the Rcs phosphorelay led to the smaller cell size of *Y. enterocolitica*. The Δ*rcsF*, Δ*rcsC*, and Δ*rcsB* single mutants were 24, 17, and 27% smaller, respectively, compared to the wild-type strain (Figure 6 and Supplementary Figure 4). Thus the cell size of the double mutant strains was further reduced compared with the Δ*opgGH* single mutant. The cell size of the Δ*opgGH*-Δ*rcsF*, Δ*opgGH-*Δ*rcsC*, and Δ*opgGH-*Δ*rcsB* double mutants were 17, 12, and 23%, respectively, smaller than those of the Δ*opgGH* single mutant (Figure 6 and Supplementary Figure 4).

### Relationship Between OPGs and the Rcs Phosphorelay According to the Expression of RcsB-Regulated Genes in *Y. enterocolitica*

**FIGURE 7:**
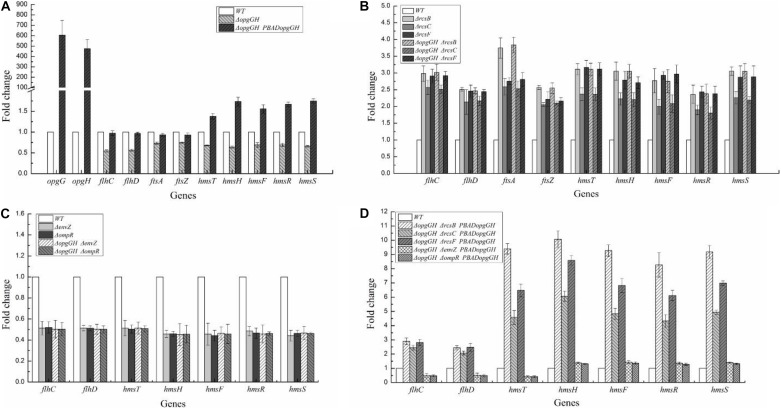
Transcriptional changes in the mutant strains of *Y. enterocolitica*. *Y. enterocolitica* grown to the mid-log phase in LNNS medium and total RNA was extracted. Expression of *opgGH*, *flhDC*, *ftsAZ*, *hmsHFRS*, and *hmsT* were determined by RT-qPCR *in vitro*. The 16S rRNA gene was used as a normalization control. **(A)** Gene transcription in Δ*opgGH* and Δ*opgGH*-P_*BAD*_*opgGH* strains. **(B)** Gene transcription in Δ*rcsF*, Δ*rcsC*, Δ*rcsB*, Δ*opgGH-*Δ*rcsF*, Δ*opgGH-*Δ*rcsC*, and Δ*opgGH-*Δ*rcsB* mutant strains. **(C)** Gene transcription in Δ*envZ*, Δ*ompR*, Δ*opgGH-*Δ*envZ*, and Δ*opgGH-*Δ*ompR* mutant strains. **(D)** Gene transcription in Δ*opgGH-*Δ*rcsF*-P_*BAD*_*opgGH*, Δ*opgGH*-Δ*rcsC-*P_*BAD*_*opgGH*, Δ*opgGH-*Δ*rcsB*-P_*BAD*_*opgGH*, Δ*opgGH-*Δ*envZ-*P_*BAD*_*opgGH*, and Δ*opgGH-*Δ*ompR-*P_*BAD*_*opgGH* strains. The data are presented as the mean ± SD of at least three independent experiments.

The above data indicate that the reduced motility, biofilm formation and c-di-GMP production caused by OPGs deficiency could be restored by disruption of the Rcs phosphorelay. Furthermore, OPGs deficiency could not affect these phenotypes in the absence of Rcs phosphorelay in *Y. enterocolitica*. These results provide evidence that loss of OPGs synthesis decreased *Y. enterocolitica* motility, biofilm formation and c-di-GMP production through acting on the Rcs phosphorelay. In *D. dadantii*, the Rcs phosphorelay regulates the *flhDC* and *ftsAZ* operons required for the expression of flagellar apparatus genes and cell division, respectively (Bontemps-Gallo et al., 2013). Furthermore, Rcs signaling controls the transcription of genes involved in biofilm formation: *hmsT* encoding diguanylate cyclase essential for c-di-GMP biosynthesis and *hmsHFRS* required for biosynthesis of poly-β-1,6-*N*-acetylglucosamine exopolysaccharide (EPS) (Fang et al., 2015). In this study, we analyzed transcription levels of these genes to determine whether OPGs deficiency activated the Rcs phosphorelay in *Y. enterocolitica*. The results revealed that all these genes were repressed in the absence of OPGs biosynthesis. Thus, the expression of *flhDC*, *hmsT, hmsHFRS*, and *ftsAZ* in the Δ*opgGH* strain was downregulated by an average of 45, 33, 34, and 27%, respectively, but the expression of all these genes in the Δ*opgGH*-P_*BAD*_*opgGH* strain was restored when compared to the wild-type strain; furthermore, the levels of *hmsT* and *hmsHFRS* exceeded those in the wild-type strain by an average of 1.38- and 1.68-fold (Figure 7A).

However, expression levels of genes downregulated by OPGs deficiency were increased in all double-mutant strains. The transcription of *flhDC*, *hmsT*, *hmsHFRS*, and *ftsAZ* in Δ*opgGH-*Δ*rcsF*/Δ*opgGH-*Δ*rcsC*/Δ*opgGH-*Δ*rcsB* was upregulated by an average of 2.68-fold/2.34-fold/2.74-fold, 3.11-fold/2.36-fold/3.10-fold, 2.74-fold/2.07-fold/2.81-fold, and 2.48-fold/2.32-fold/3.19-fold, respectively, when compared to the wild-type strain (Figure 7B). The deletion of *rcsF/rcsC/rcsB* also resulted in increase in these genes expression, however, there was no significant change between Δ*opgGH-*Δ*rcsF/*Δ*opgGH-*Δ*rcsC/*Δ*opgGH-*Δ*rcsB* double mutant and Δ*rcsF/*Δ*rcsC/*Δ*rcsB* single mutant in the transcription of *flhDC*, *hmsT*, *hmsHFRS*, and *ftsAZ* (*P* > 0.05) (Figure 7B). All these results indicated that that deletion of any Rcs phosphorelay gene reversed transcriptional changes caused by the lack of OPGs.

### Disruption of the EnvZ/OmpR Phosphorelay Further Reduced Motility, c-di-GMP Production, and Biofilm Formation in the *Y. enterocolitica* Δ*opgGH* Mutant

It was shown that defects in motility caused by OPGs deficiency could be restored by disrupting the *envZ*-*ompR* operon in *E. coli* (Fiedler and Rotering, 1988). In this study, we constructed Δ*opgGH-*Δ*envZ* and Δ*opgGH-*Δ*ompR* double mutants to test the effect of EnvZ/OmpR phosphorelay on motility and *flhDC* expression in the *Y. enterocolitica* Δ*opgGH* mutant. However, as described previously, OmpR directly, positively regulates the expression of *flhDC* in *Y. enterocolitica*, as well as in *Y. pseudotuberculosis* (Hu et al., 2009; Raczkowska et al., 2011; Meng et al., 2019). The swim diameter and *flhDC* expression decreased by 47 and 49%, respectively, due to the inactivation of *envZ* or *ompR* (Figures 3, 7C). Thus both the Δ*opgGH-*Δ*envZ* and Δ*opgGH-*Δ*ompR* strains reduced swim motility and *flhDC* expression by 46 and 50%, respectively, when compared to the wild-type strain (Figures 3, 7C). In contrast to *E. coli*, disruption of the EnvZ/OmpR phosphorelay in *Y. enterocolitica* did not recover motility decreased by OPGs deficiency.

In addition, the results revealed that inactivation of *envZ* or *ompR* markedly decreased both biofilm formation and c-di-GMP production in the Δ*opgGH* strain. After 72 h of incubation, both Δ*opgGH-*Δ*envZ* and Δ*opgGH-*Δ*ompR* double mutants were decreased by 21% in biofilm formation when compared to the Δ*opgGH* single mutant (Figure 4). The intracellular c-di-GMP production in Δ*opgGH-*Δ*envZ* and Δ*opgGH-*Δ*ompR* double mutants were 54 and 56% reduction, respectively, compared to the Δ*opgGH* single mutant (Figure 5). Correspondingly, the transcription of the *hmsT* and *hmsHFRS* were downregulated by an average of 50 and 54% when compared to the wild-type strain (Figure 7C).

### Rcs and EnvZ/OmpR Phosphorelays Showed Opposite Effects on OPGs-Induced Biofilm and c-di-GMP Production in *Y. enterocolitica*

In this study, it was shown that *Y. enterocolitica* was capable of forming biofilms in LNNS medium and the increase in OPGs greatly induced biofilm formation and c-di-GMP production (Figures 4, 5 and Supplementary Figure 3). Since the inactivation of Rcs phosphorelay upregulated the *flhDC*, *hmsT*, and *hmsHFRS* expression which is responsible for biofilm and c-di-GMP production (Figure 7B), we further investigated the effect of Rcs phosphorelay on OPGs-induced biofilm formation and c-di-GMP synthesis by transfecting double mutants with pBAD24-*opgGH* to create Δ*opgGH-*Δ*rcsF*-P_*BAD*_*opgGH*, Δ*opgGH-*Δ*rcsC-*P_*BAD*_*opgGH* and Δ*opgGH-*Δ*rcsB-*P_*BAD*_*opgGH* strains. As a result, the Δ*opgGH-*Δ*rcsF*-P_*BAD*_*opgGH*, Δ*opgGH-*Δ*rcsC-*P_*BAD*_*opgGH*, and Δ*opgGH-*Δ*rcsB-*P_*BAD*_*opgGH* strains had increased swim diameter by 31, 12, and 35%, respectively, compared to the Δ*opgGH*-P_*BAD*_*opgGH* strain (Figure 3). The biofilm formation in Δ*opgGH-*Δ*rcsF*-P_*BAD*_*opgGH*, Δ*opgGH-*Δ*rcsC-*P_*BAD*_*opgGH*, and Δ*opgGH-*Δ*rcsB-*P_*BAD*_*opgGH* strains were 86, 68, and 1.15-fold increase, respectively (Figure 4); correspondingly, in these strains, the c-di-GMP production was increased by 96, 48% and 1.27-fold when compared to the Δ*opgGH*-P_*BAD*_*opgGH* strain (Figure 5). The expression of the *flhDC*, *hmsT*, and *hmsHFRS* genes in Δ*opg GH-*Δ*rcsF*-P_*BAD*_*opgGH*/Δ*opgGH-*Δ*rcsC-*P_*BAD*_*opgGH*/Δ*opgGH-*Δ*rcsB-*P_*BAD*_*opgGH* was upregulated by an average of 2.64-fold/2.26-fold/2.68-fold, 6.48-fold/4.58-fold/9.39-fold, and 7.13-fold/5.04-fold/9.20-fold, respectively, when compared to the wild-type strain (Figure 7D). These results indicated that disruption of the Rcs phosphorelay increased motility and further promoted induction of biofilm and c-di-GMP production by OPGs in Δ*opgGH-*P_*BAD*_*opgGH* strain.

We also investigated the relationship between OPGs overproduction and the EnvZ/OmpR phosphorelay using the same approach, i.e., transforming Δ*opgGH-*Δ*envZ* and Δ*opgGH-*Δ*ompR* double mutants with pBAD24-*opgGH*. Although OPGs overproduction upregulated c-di-GMP and biofilm production in Δ*opgGH*-Δ*envZ-*P_*BAD*_*opgGH* and Δ*opgGH-*Δ*ompR-*P_*BAD*_*opgGH* strains compared to the double mutants, these effects were significantly weaker than those in the Δ*opgGH*-P_*BAD*_*opgGH* strain (Figures 4, 5 and Supplementary Figure 3). Both the Δ*opgGH*-Δ*envZ-*P_*BAD*_*opgGH* and Δ*opgGH-*Δ*ompR-*P_*BAD*_*opgGH* strains had decreased swim diameter by 52% compared to the Δ*opgGH*-P_*BAD*_*opgGH* strain (Figure 3). The biofilm formation was also decreased by 39 and 38%, respectively (Figure 4); accordingly, the c-di-GMP production was decreased by 58 and 57% when compared to the Δ*opgGH*-P_*BAD*_*opgGH* strain (Figure 5). The expression of the *flhDC* and *hmsT* genes in Δ*opgGH*-Δ*envZ-*P_*BAD*_*opgGH*/Δ*opgGH-*Δ*ompR-*P_*BAD*_*opgGH* strains was downregulated by an average of 50%/51% and 58%/59%, respectively, when compared to the wild-type strain (Figure 7D). Though OPGs overproduction upregulated the *hmsHFRS* expression in Δ*opgGH*-Δ*envZ*-P_*BAD*_*opgGH* and Δ*opgGH*-Δ*ompR*-P_*BAD*_*opgGH* strains compared to the wild-type strain, the expression was lower than those in the Δ*opgGH*-P_*BAD*_*opgGH* strain (Figures 7A,D). All these results indicated that the disruption of the EnvZ/OmpR phosphorelay decreased motility and prevented the induction of biofilm and c-di-GMP production by OPGs in Δ*opgGH-*P_*BAD*_*opgGH* strain.

## Discussion

Osmoregulated periplasmic glucans are important signaling molecules existing in the periplasmic space of many Gram-negative bacteria, where they participate in environmental perception and regulation of genes involved in virulence of *Enterobacteriaceae* pathogens (Bontemps-Gallo and Lacroix, 2015; Bontemps-Gallo et al., 2017). However, the role of OPGs in *Y. enterocolitica* was previously unknown. Results in this study indicate that deletion of *opgGH* operon in the chromosome of *Y. enterocolitica* decreased motility, c-di-GMP production, biofilm formation and cell size of the bacteria, indicating that OPGs regulate several functions and confer growth advantage to *Y. enterocolitica*. In addition to the presence in the periplasmic space, OPGs are also secreted into the extracellular space, where they can directly interact with antibiotics, thus preventing their cell-damaging effects (Bontemps-Gallo and Lacroix, 2015). Previous findings suggest that OPGs are critical for the tolerance of *P. aeruginosa* to aminoglycoside antibiotics by sequestering antibiotic molecules away from their cellular targets only when the bacteria were in a biofilm (Mah et al., 2003). In this study, the effect of OPGs on antibiotic sensitivity was performed in planktonic-grown *Y. enterocolitica*, and it was found that the OPGs of wild-type strain did not confer resistance to antibiotics, which was similar to that of in *P. aeruginosa*. However, the overexpression of *opgGH* resulted in more than twofold increase in OPGs production compared to the wild-type strain (Figure 2B), which enhanced *Y. enterocolitica* tolerance to chloramphenicol and polymyxin B with different mechanisms of action. Considering that OPGs can be secreted extracellularly, it can be easily assumed that the Δ*opgGH-*P_*BAD*_*opgGH* strain produced so much OPGs, leading to a leakage of OPGs into the external environment. Thus, the phenotype observed is the consequence of OPGs overproduction but does not indicate that OPGs are involved in antibiotic resistance in *Y. enterocolitica*.

Recent study reported that *Y. pestis* lost the *opgGH* operon during its emergence from the enteropathogen *Y. pseudotuberculosis*. *Y. pseudotuberculosis* did not produce OPGs even if the *opgGH* operon was expressed. Inactivation of the *opgGH* operon showed normal motility, biofilm formation, resistance to polymyxin B and virulence (Quintard et al., 2015). Furthermore, *Y. pestis* re-introducing *opgGH* grew normally and was able to complete its infectious cycle (flea-host) (Quintard et al., 2015). Data in this study showed that OPGs can be detected in *Y. enterocolitica* under low-nutrient low-salt conditions and that deletion of the *opgGH* operon generated a pleiotropic phenotype and OPGs overproduction conferred *Y. enterocolitica* higher resistance to polymyxin B. NCBI homologous alignment revealed that *opgG* and *opgH* nucleotide between the two species shows 81.75 and 80.26% identities over the whole sequence respectively, indicating the difference in nucleotide sequences of *opgGH* between the two species, which help to explain why *opgGH* in *Y. enterocolitica* encodes functional OPGs, but *opgGH* in *Y. pseudotuberculosis* had no effect of this. Or as explained by Quintard et al. (2015) that *Y. pseudotuberculosis* might produce OPGs under particular conditions or might lack an essential factor for OPGs biosynthesis, since the *opgGH* operon from *Y. pseudotuberculosis* could encode functional OPGs in *E. coli* and *D. dadantii* (Quintard et al., 2015). However, inactivation of *opgGH* reduced the cell size in both *Y. enterocolitica* and *Y. pseudotuberculosis.*

The Rcs phosphorelay is an atypical TCS conserved in *Enterobacteriaceae* that allows bacteria to perceive external environment stimuli and modify gene expression (Clarke, 2010; Guo and Sun, 2017); however, its functional activity in *Y. enterocolitica* has not been reported. In the present work, we found that the *flhDC*, *hmsT*, *hmsHFRS*, and *ftsAZ* genes expression regulated by Rcs phosphorelay were repressed by OPGs deficiency, but deletion of *rcsF*, *rcsC*, and *rcsB* encoding Rcs phosphorelay components in Δ*opgGH* mutant restored expression levels of *flhDC*, *hmsT*, *hmsHFRS* and *ftsAZ*, as well as cell motility, c-di-GMP production and biofilm formation in *Y. enterocolitica*. In fact, the increased *flhDC*, *hmsT*, *hmsHFRS*, and *ftsAZ* were also observed in Δ*rcsF*, Δ*rcsC*, and Δ*rcsB* single mutants cultured in LNNS medium, suggesting that in *Y. enterocolitica*, the Rcs system is activated under low-nutrient low-salt conditions, which may explain why deletion of *rcsF*/*rcsC*/*rcsB* not only rescued but further promoted motility, c-di-GMP production and biofilm formation in the Δ*opgGH* mutant. However, there was no significant difference between Δ*opgGH-*Δ*rcsF/*Δ*opgGH-*Δ*rcsC/*Δ*opgGH-*Δ*rcsB* double mutant and Δ*rcsF/*Δ*rcsC/*Δ*rcsB* single mutant in terms of the expression level of all these genes, suggesting that OPGs deficiency could not affect these genes’ expression in the inactivation of Rcs phosphorelay in *Y. enterocolitica*. All these results provide evidence that downregulation of OPGs synthesis decreases *Y. enterocolitica* motility, c-di-GMP production and biofilm formation through activation of the Rcs phosphorelay. In addition, it has been reported that the Δ*opgGH* mutant decreased swim diameter by 70% and lost the capacity to form a biofilm in *D. dadantii* (Bouchart, 2006; Bontemps-Gallo et al., 2013), while in this study the swim diameter and biofilm formation only decreased by 21 and 24%, respectively, in LNNS medium but the results can be reproduced. It was suspected that this small change may be related to the over-activation of the Rcs system in the wild-type of *Y. enterocolitica* under low-nutrient low-salt conditions, thus even if the OPGs deficiency leads to activation of the Rcs system, the phenotypic changes in the response were not so obvious.

**FIGURE 8:**
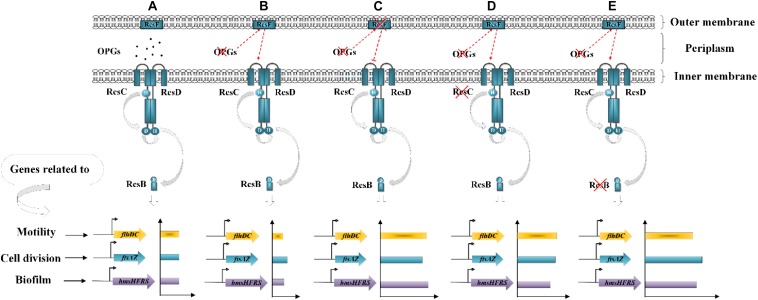
Working model of the relationship between OPGs and the Rcs phosphorelay in *Y. enterocolitica*. **(A)** In the wild-type strain, the Rcs system represses cell motility, division and biofilm formation by inhibiting *flhDC*, *ftsAZ*, and *hmsHFRS* expression. **(B)** OPGs deficiency activates the Rcs phosphorelay and inhibits *flhDC, ftsAZ*, and *hmsHFRS* expression, resulting in further repression of cell motility, division, and biofilm formation. **(C–E)** Disruption of the Rcs phosphorelay pathway downregulates RcsB phosphorylation, restoring cell motility, division, and biofilm formation by enhancing the expression of *flhDC*, *ftsAZ*, and *hmsHFRS*.

Based on the expression of RcsB-regulated operons, we propose a model linking OPGs to the Rcs phosphorelay in *Y. enterocolitica* (Figure 8). Combined the previously reported state of phosphorylation of RcsB in the wild-type of *D. dadantii* (Madec et al., 2014), we can propose that: in the wild-type *Y. enterocolitica* (Figure 8A), OPGs are synthesized and RcsB is phosphorylated at a certain level providing certain repression of *flhDC*, *hmsHFRS* and *ftsAZ*, whereas in the absence of OPGs synthesis (Figure 8B), the activation of the Rcs phosphorelay is induced, leading to decrease in motility, biofilm formation and cell division through downregulation of *flhDC*, *hmsHFRS*, and *ftsAZ*. In the absence of both OPGs and Rcs signaling (Figures 8C–E), when RcsB is not phosphorylated, motility, biofilm formation and cell division are restored through upregulation of *flhDC, hmsHFRS*, and *ftsAZ*. This model characterizes the relationship between OPGs and the Rcs phosphorelay illustrating an important role of OPGs in *Y. enterocolitica*, which regulate the Rcs signal transduction pathway, preventing its overactivation, thus promoting cell motility, division, and biofilm formation. In this study, OPGs deficiency could not affect the expression of these genes in the absence of RcsF, indicating that the RcsF protein is required for the perception of OPGs defect in *Y. enterocolitica*. It should be noted that RcsF-dependent activation of the Rcs phosphorelay is a consequence of BamA (the core protein for β-barrel assembly) failing to bind RcsF and funnel it to OmpA (the β-barrel) (Cho et al., 2014). However, it still remains to be elucidated whether this mechanism works for *Y. enterocolitica* when Rcs system is activated by loss of OPGs.

In *Y. enterocolitica*, Δ*rcsB* single mutant upregulated the *ftsAZ* operon, indicating that the *ftsAZ* genes are repressed by RcsB, which is in contrast with previous findings that the *ftsAZ* operon was activated by RcsB in *E. coli* and *Proteus mirabilis* (Howery et al., 2016). Our finding that activation of the Rcs system decreased *ftsAZ* expression is different from earlier reports showing that Rcs activation enhanced *ftsAZ* expression in *D. dadantii* (Bontemps-Gallo et al., 2013) and *E. coli* (Hill et al., 2013). These data suggest the existence of species-specific differences in the regulation patterns of the Rcs system. Previous studies have reported that OpgH regulates bacterial cell architecture by sequestering FtsZ and, thus, affecting cell length independently from OPGs synthesis (Hill et al., 2013; Quintard et al., 2015). Here, we observed reduction of *ftsAZ* expression and decrease in the cell size of the Δ*opgGH* mutant, which may be due not to the lack of OPGs or activation of the Rcs system but rather to the absence of other OpgH activities unrelated to OPGs biosynthesis. However, as FtsZ levels should be increased by about 50% to cause cell size reduction of 15–20% (Hill et al., 2013), it may be speculated that a possible reason for cell size decrease in Δ*opgGH-*Δ*rcsF*, Δ*opgGH-*Δ*rcsC*, and Δ*opgGH-*Δ*rcsB* strains could be the overexpression of *ftsAZ* (Figure 7B).

It was reported that OmpR negatively regulates the expression of *flhDC* in *E. coli*, thus defects in motility caused by OPGs deficiency could be restored by disrupting the EnvZ/OmpR system (Fiedler and Rotering, 1988). While in *Y. enterocolitica*, OmpR was shown to positively regulate the expression of *flhDC* and motility (Raczkowska et al., 2011; Meng et al., 2019). Thus, inactivation of *envZ* or *ompR* in *Y. enterocolitica* Δ*opgGH* mutant led to 46% reduction in swim motility compared to the wild-type strain, which is in agreement with a recent report in *D. dadantii* that inactivation of *envZ* or *ompR* in the *opgG* background resulted in a 40% reduction in motility relative to the wild-type strain (Caby et al., 2018). Unlike *E. coli*, inactivation of EnvZ/OmpR phosphorelay system cannot restore *flhDC* expression and motility in the OPGs-deficient strain both in *Y. enterocolitica* and *D. dadantii*, Rcs and EnvZ/OmpR phosphorelays showed the opposite effects on the regulation of *flhDC* expression and motilit**y** in *Y. enterocolitica* and *D. dadantii*. In addition, it should be noted that Rcs and EnvZ/OmpR systems control the mechanism of OPGs succinylation in *D. dadantii*, but, unlike for Rcs system, EnvZ/OmpR system is not controlled by OPGs concentration but requires OPGs for proper activation (Bontemps-Gallo et al., 2016; Caby et al., 2018).

**FIGURE 9:**
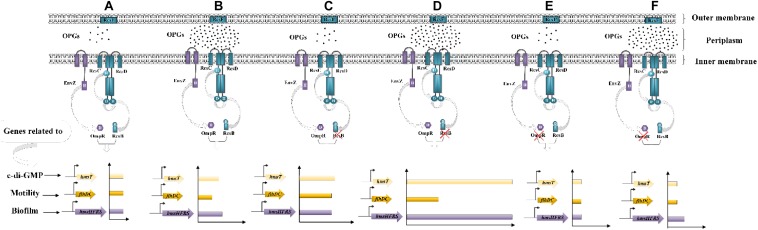
Effects of Rcs and EnvZ/OmpR phosphorelays on OPGs-induced biofilm and c-di-GMP production in *Y. enterocolitica*. **(A,B)** OPGs overproduction induces the expression of *hmsHFRS* and *hmsT*, thus promoting biofilm and c-di-GMP production in the presence of both Rcs and EnvZ/OmpR phosphorelays. **(C,D)** Disruption of the Rcs phosphorelay upregulates the expression of *flhDC*, *hmsHFRS* and *hmsT*, resulting in increased motility and further promoted the induction of biofilm and c-di-GMP production regulated by OPGs. **(E,F)** Disruption of the EnvZ/OmpR phosphorelay decreases *flhDC*, *hmsHFRS*, and *hmsT* expression, inhibiting motility and preventing the induction of biofilm and c-di-GMP production regulated by OPGs.

The ability to form biofilms is important for protection of bacteria from various stresses and critically depends on their motility (Brzostek et al., 2012; Pruss, 2017). In this work, we found that *Y. enterocolitica* was capable of forming biofilms under low-nutrient low-salt conditions and that OPGs could play an important role in promoting the process (Figure 4 and Supplementary Figure 3). The *flhDC* operon encodes the master regulator of flagellum biosynthesis and is the key factor in the initial attachment of biofilms (Raczkowska et al., 2011). The *hmsHFRS* operon is responsible for the synthesis of poly-β-1,6-*N*-acetylglucosamine EPS constituting the extracellular polymeric substance, which is a key factor in promoting biofilm maturation (Bomchil et al., 2003; Fang et al., 2015). The *hmsT* encodes diguanylate cyclase required for biosynthesis of c-di-GMP, a second messenger involved in the regulation of a variety of bacterial behaviors, including biofilm formation (Fang et al., 2015). In this study, analysis of *flhDC*, *hmsHFRS*, and *hmsT* expression revealed a mechanism underlying the effect of Rcs and EnvZ/OmpR phosphorelays on OPGs-induced biofilm formation (Figure 9). OPGs overproduction in the periplasm promoted biofilm and c-di-GMP production in the presence of both phosphorelays by inducing the expression of *hmsHFRS* and *hmsT* genes (Figure 9B). Inactivation of the Rcs phosphorelay upregulated the *flhDC*, *hmsHFRS*, and *hmsT* expression, leading to the increased motility and further promoted the induction of biofilm and c-di-GMP production regulated by OPGs (Figure 9D); in contrast, inactivation of the EnvZ/OmpR phosphorelay downregulated the *flhDC*, *hmsHFRS* and *hmsT* expression, resulting in decreased motility and prevented the induction of biofilm and c-di-GMP production regulated by OPGs (Figure 9F). Thus, Rcs and EnvZ/OmpR phosphorelays exert the opposite effects on the regulation of OPGs-induced c-di-GMP and biofilm production in *Y. enterocolitica*. A recent study showed that *Pantoea alhagi* senses environmental osmolarity changes through the EnvZ/OmpR system and LrhA to regulate synthesis of OPGs, EPS production and flagella-dependent motility. OPGs control the Rcs activation in a concentration-dependent manner, and a high concentration of OPGs induced by increased medium osmolarity is maintained to achieve the high level of activation of the Rcs phosphorelay which results in enhanced EPS synthesis and decreased motility in *P. alhagi* (Li et al., 2019). As motility and biofilm formation are critical for bacterial colonization of the host, these findings indicate that OPGs associated with Rcs and EnvZ/OmpR systems play a key role in shaping the pathogenic phenotype of bacteria. Further studies are required to fully understand the mechanism underlying OPGs-mediated biofilm formation in *Y. enterocolitica*, which is likely multi-factorial.

## Conclusion

This study shows that OPGs are a part of the Rcs signal transduction pathway in *Y. enterocolitica*, regulating gene expression and producing a pleiotropic phenotype by disturbing the Rcs system. OPGs enhance antibiotic resistance of *Y. enterocolitica*; furthermore, they promote cell motility, c-di-GMP biosynthesis and biofilm production in low-nutrient low-salt conditions. It was also found that Rcs and EnvZ/OmpR phosphorelays exert the opposite effects on the regulation of OPGs-induced c-di-GMP and biofilm production in *Y. enterocolitica*. These findings reveal part of the biological function of OPGs in *Y. enterocolitica* and its relationship with Rcs and EnvZ/OmpR systems, which is important for understanding complex mechanisms underlying *Y. enterocolitica* pathogenicity.

## Data Availability Statement

All datasets generated for this study are included in the article/Supplementary Material.

## Author Contributions

JM performed the experiments under the guidance of JC. JC and JM developed the idea for the study and designed the research. JM, CH, and XH analyzed the experimental data and drafted the manuscript. DL and BH made substantial contributions to conception, interpretation of data, and revised the manuscript. All authors read and approved the final manuscript.

## Conflict of Interest

The authors declare that the research was conducted in the absence of any commercial or financial relationships that could be construed as a potential conflict of interest.
