# Supplementary material for: Osmoregulated Periplasmic Glucans Transmit External Signals Through Rcs Phosphorelay Pathway in Yersinia enterocolitica
Source: Front Microbiol. 2020 Feb 5;11:122. doi: 10.3389/fmicb.2020.00122 (PMC7013093; doi:10.3389/fmicb.2020.00122)
Supplement: Supplementary file 1 [file Table_1.DOCX]

Supplementary Materials

## Supplementary Figures


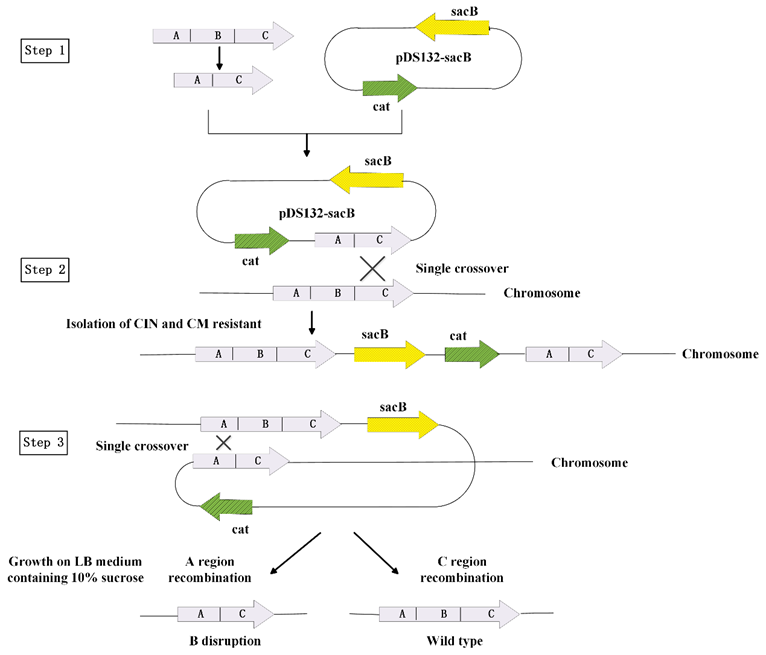


**Supplementary Figure 1.** Strategy for gene deletion in *Y. enterocolitica* based on the two-step homologous recombination procedure. In the first step, the suicide plasmids containing the *cat-sacB* cassette flanked by homologous sequence were transformed into *Y. enterocolitica* chromosome by homologous recombination. Transformants were screened on LB agar containing chloramphenicol and CIN antibiotics (for screening of *Y. enterocolitica*) composed of cefsulodin, irgasan and novobiocin, and screened by PCR for the presence of *sacB* gene. Sucrose sensitivity of selected clones were confirmed by patching onto LB agar containing 10% sucrose. Subsequently, deletion constructs were used to transform appropriate insertion-deletion mutants in order to remove the *cat-sacB* cassette. Transformants were plated on LB agar containing 10% sucrose and mutants were verified by PCR and further confirmed by gene sequencing.


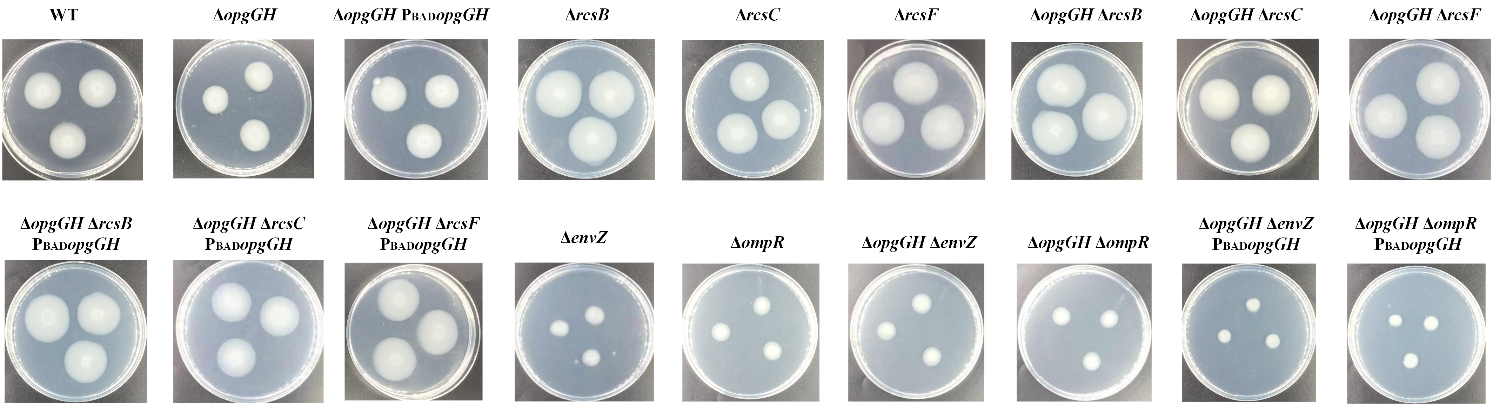


**Supplementary Figure 2.** Changes in swim motility of wild-type and mutant strains in LNNS medium. Images of swim plates were captured after 48 h of incubation.


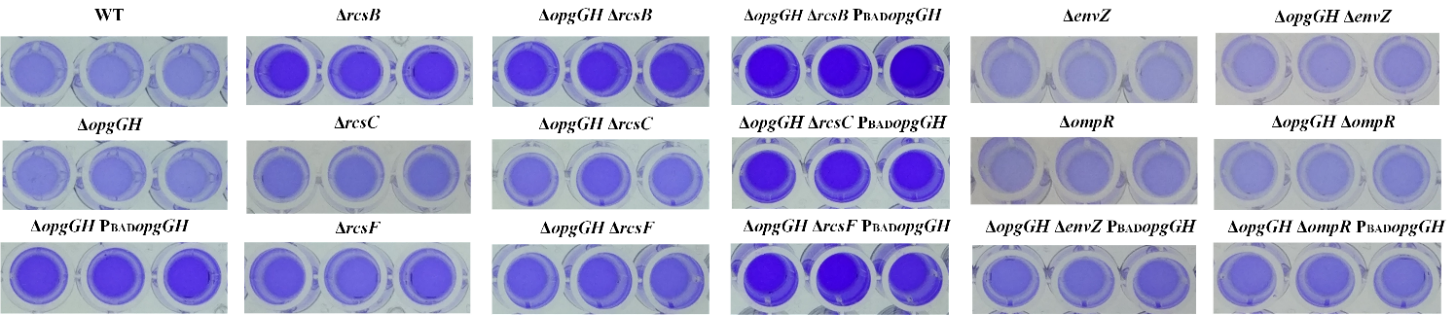


**Supplementary Figure 3.** Images of purple color depth after released the biofilms attached with crystal violet dye with ethanol-acetone solution in wild-type and mutant strains. After 72 h of incubation, the formed biofilms were washed twice with PBS and stained with crystal violet staining solution (0.1%). Wells were washed with water and treated with ethanol-acetone solution to release the dye absorbed in the biofilm.


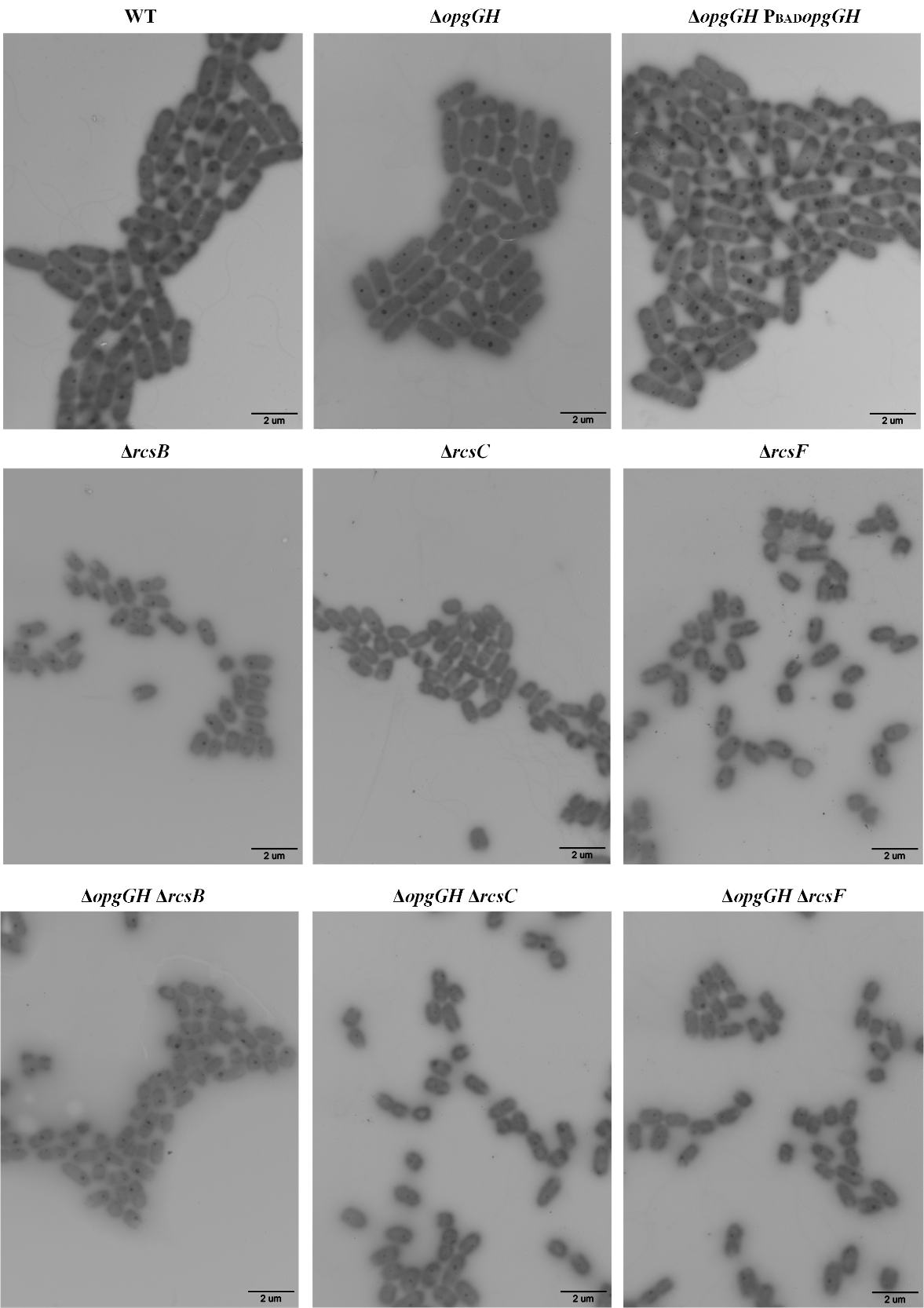


**Supplementary Figure 4.** Transmission electron microscopy images of wild-type and mutant strains. Bacteria grown to the mid-log phase in LNNS medium were immobilized on 200-mesh copper grids and fixed with 0.5% glutaraldehyde. And the cell length was visualized using a JEM-1230 transmission electron microscopy at a magnification of 10,000x for all strains.

**Supplementary Tables**

**Supplementary Table 1.** Primers used for strain and plasmid construction in this study.

| Primer name | Primer sequence (5′ 3′) |
| --- | --- |
| *opgGH-up-F* | ACTGCATGCATGGATGAAATCATTCAATAGG |
| *opgGH-up-R*  *opgGH-down-F* | TCTATTGACGTTCGACAAAATATTAACTCCCACAT  ATGTGGGAGTTAATATTTTGTCGAACGTCAATAGACGGTATGTAAAAATAGAAAAAC |
| *opgGH-down-R* | ACTGCGAGCTCCAGAAAAACACAGGCCTCGGC |
| *p-opgGH-F* | ACTGGTCGACACATATTCGCTAGTTGTTATCG |
| *p-opgGH-R* | ATTGAAGCTTCTATTTCGCGGCGGGGAGGGCCTT |
| *rcsB-up-F* | ACTGCATGCAGAAGTGCGTTCTATAATCACA |
| *rcsB-up-R* | GGTTATGGGTTACCTTGCTGCAACAGTCTGGTGAC |
| *rcsB-down-F* | GTCACCAGACTGTTGCAGCAAGGTAACCCATAACCCTTCTGTTCCCCAACCCGGCAT |
| *rcsB-down-R* | ACTGCGAGCTCATCTGGATGAGAATGCAGATC |
| *rcsC-up-F* | ACTGCATGCCTCAATGGCGACGATCGGGTTA |
| *rcsC-up-R* | TCTGATACCATGATGAAAGCATCGCCGGGCAGGCT |
| *rcsC-down-F* | AGCCTGCCCGGCGATGCTTTCATCATGGTATCAGAAGCAGAAATAGAAATATATACC |
| *rcsC-down-R* | ACTGCGAGCTCCAGATTTAGCCATAATAGTAC |
| *rcsF-up-F* | ACTGCATGCAAATCATTGGAAGAACTGCAAC |
| *rcsF-up-R* | AGAAATTCCTCAGCCTTTTTATTTAAGAAGCGCTA |
| *rcsF-down-F* | TAGCGCTTCTTAAATAAAAAGGCTGAGGAATTTCTGTGTGTTTTCCTTTAATCAGAT |
| *rcsF-down-R* | ACTGCGAGCTCCTTTGCGGTAGGCTGGGCGTG |
| *envZ-up-F* | ACTGCATGCGATGGATCGCTTGCTAACTCGT |
| *envZ--up-R*  *envZ-down-F* | GCTTTACTGCCGTCCGGCACAAATACGTAGCCCA  TGGGCTACGTATTTGTGCCGGACGGCAGTAAAGCGTTTTATTTTGGTTATTTAAT |
| *envZ -down-R* | ACTGCGAGCTCTGGTGATTAGGTTGTTCGGTG |
| *ompR-up-F* | ACTGCATGCATAACCCGCGAGGGCTGGCAG |
| *ompR-up-R* | TTTATTACTCCCAAAGGCCGTATTGCCCGAATCTA |
| *ompR-down-F* | TAGATTCGGGCAATACGGCCTTTGGGAGTAATAAAGGCGATGGCGCTTTTCTCCACG |
| *ompR-down-R* | ACTGCGAGCTCGAATGATGCCCTTACCGACCTG |

Restriction sites are underlined.

**Supplementary Table 2**. Primers used in the RT-qPCR assay.

| Primer name | Primer sequence (5′ 3′) | Amplicon size (bp) |
| --- | --- | --- |
| *q-opgG-F* | CGGCAGATGAAACCAACGAT | 101 |
| *q-opgG-R* | GGTGAAATGCAGGCGGTAAT |  |
| *q-opgH-F* | ATTGGCTTCGGTCAATGCTC | 83 |
| *q-opgH-R* | CTTCGTCGTCTTGTGCCAAT |  |
| *q-flhC-F* | GGACCTTGGTTCGCTTTGTT | 170 |
| *q-flhC-R* | GGCAGATTGCGGAGAAAGTT |  |
| *q-flhD-F* | CCTCAGCGATGTTTCGTCTC | 176 |
| *q-flhD-R* | CTGCAAGTCATCCACACGAG |  |
| *q-ftsA-F* | GCTGTTGGGTTGTTGCACTA | 108 |
| *q-ftsA-R* | CAGCCAGCTATTGATGCGTT |  |
| *q-ftsZ-F* | ATTTGCATCCGACAATGCGA | 171 |
| *q-ftsZ-R* | TGCTGGTAACGGTGATCCAT |  |
| *q-hmsT-F* | TATAATCGCCGTGGGTTGGA | 144 |
| *q-hmsT-R* | CACTAAGGCTTGGTCTCCCA |  |
| *q-hmsH-F* | GCGCCGGTCCTTGATTATTT | 82 |
| *q-hmsH-R* | CGATTTGCAGCCAGTCATCA |  |
| *q-hmsF-F* | TGGCAATGCCGTTAATGGAG | 144 |
| *q-hmsF-R* | CCTCTGGTTTGGTCCAGTCT |  |
| *q-hmsR-F* | GATGATGTACCGCCTCCAGA | 96 |
| *q-hmsR-R* | GTGAATAGTTTCCCGCGCAT |  |
| *q-hmsS-F* | AGCATGGAAATGACGGGAGA | 134 |
| *q-hmsS-R* | TTAACGACCGGTGCAACTTC |  |
| *16S rRNA-F* | GCACGTAATGGTGGGAACTC | 183 |
| *16S rRNA-R* | CTCCAATCCGGACTACGACA |  |
